# Late emergence of A594V and L595W mutations related to ganciclovir resistance in a patient with HCMV retinitis and long-term HIV progression

**DOI:** 10.1590/1414-431X20154507

**Published:** 2015-07-04

**Authors:** S.N. Slavov, F.C. Vilar, V.M.D. Wagatsuma, R.C. Santana, A.A. Machado, B.A.L. da Fonseca, S. Kashima, D.T. Covas

**Affiliations:** 1 Hemocentro de Ribeirão Preto, Faculdade de Medicina de Ribeirão Preto, Universidade de São Paulo, Ribeirão Preto, SP, Brasil; 2 Divisão de Moléstias Infecciosas e Tropicais, Departamento de Clínica Médica, Faculdade de Medicina de Ribeirão Preto, Universidade de São Paulo, Ribeirão Preto, SP, Brasil; 3 Laboratório de Hematologia Experimental, Departamento de Clínica Médica, Faculdade de Medicina de Ribeirão Preto, Universidade de São Paulo, Ribeirão Preto, SP, Brasil; 4 Departamento de Análises Clínicas, Toxicológicas e Bromatológicas, Faculdade de Ciências Farmacêuticas de Ribeirão Preto, Universidade de São Paulo, Ribeirão Preto, SP, Brasil; 5 Departamento de Clínica Médica, Faculdade de Medicina de Ribeirão Preto, Universidade de São Paulo, Ribeirão Preto, SP, Brasil

**Keywords:** Human cytomegalovirus, HCMV, Ganciclovir, Resistance, AIDS

## Abstract

The emergence of ganciclovir (GCV) resistance during the treatment of human cytomegalovirus (HCMV) infection is a serious clinical challenge, and is associated with high morbidity and mortality. In this case report, we describe the emergence of two consecutive mutations (A594V and L595W) related to GCV resistance in a patient with HCMV retinitis and long-term HIV progression after approximately 240 days of GCV use. Following the diagnosis of retinitis, the introduction of GCV did not result in viral load reduction. The detected mutations appeared late in the treatment, and we propose that other factors (high initial HCMV load, previous GCV exposure, low CD4^+^ cell count), in addition to the presence of resistance mutations, may have contributed to the treatment failure of HCMV infection in this patient.

## Introduction

Until the 1990s, human cytomegalovirus (HCMV) was regarded as a major opportunistic virus in patients with AIDS, and was associated with high morbidity and mortality (1). The main clinical presentation of HCMV infection in patients with AIDS, HCMV-induced retinitis, was a common clinical finding (2). However, after 1996, the frequency of HCMV-retinitis in AIDS patients decreased because of the introduction of the ganciclovir (GCV) eye implant (3,4), and the use of protease and non-nucleotide reverse-transcriptase inhibitors in antiretroviral (ART) regimens worldwide (1). Despite the fact that nowadays treatment of HCMV infection is usually successful, it continues to represent a significant proportion of cases with end-organ disease in patients with AIDS (5). HCMV infection treatment failure could involve risk factors such as profound immunosuppression, previous exposure to anti-HCMV drugs, or persistent low-level HCMV replication (6). Nevertheless, retinitis relapse in patients with AIDS is mostly a result of the development of HCMV GCV resistance caused by prolonged treatment periods (2) and low adherence to ART. GCV resistance of HCMV is confined to mutations in the viral phosphotransferase gene (*UL97*) (7-9) and, less frequently, in the DNA polymerase gene (*UL54*) (10). The emergence of resistant HCMV strains presents a clinical challenge for the management of patients with AIDS, and has been associated with increased mortality (11).

Here, we report the management of a long-term HIV-progressor with simultaneous emergence of two consecutive GCV-related mutations in the *UL97* gene of HCMV following ∼240 days of GCV use for treatment of retinitis. Based on the experience acquired with this case, we propose that a combination of factors, including viral and host characteristics, is crucial for managing HCMV infection in AIDS patients.

## Case report

A 53-year-old female patient living with HIV infection for over 20 years (despite low adherence to ART) was admitted several times to the AIDS Unit of the Hospital das Clínicas da Faculdade de Medicina de Ribeirão Preto, Universidade de São Paulo, Ribeirão Preto, SP, Brazil. The first evidence of HCMV infection was registered in October 2009, when she presented with chronic diarrhea, fever, and anemia (hemoglobin=7.9 g/dL). An HCMV pp65 antigenemia test demonstrated 90 infected cells/2×10^5^ leukocytes. Because of the elevated number of pp65-positive cells, the patient was treated with intravenous GCV (10 mg/kg daily) for 21 days. By the end of the treatment period, the patient presented a CD4^+^ cell count of 65 cells/mm^3^ and an HIV load of 15,473 copies/mL.

In July 2010, the patient developed pulmonary tuberculosis but was considered cured following a 6-month treatment with 600 mg/day rifampcin, 300 mg/day isoniazid, 1.5 g/day pyrazinamide, and 1.2 mg/day ethambutol. In October of the same year, ART (300 mg/day tenofovir, 300 mg/day lamivudine, 600 mg/day efavirenz) was initiated. This treatment did not improve the patient's immunologic condition, as the CD4^+^ cell count remained very low (19 cells/mm^3^), with an HIV load of 306,771 copies/mL.

**Figure 1:**
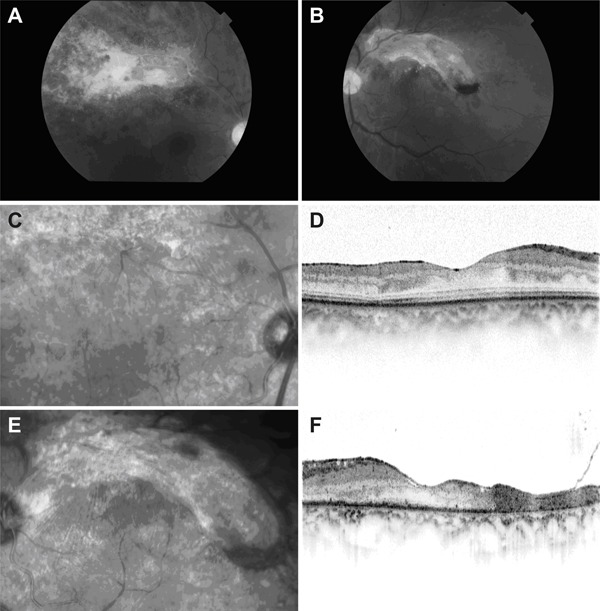
Bilateral cytomegalovirus retinitis of the patient with AIDS. *A*, Retinitis in the process of cicatrization with hemorrhage in the superior temporal arcade of the right eye. *B*, Cicatrization of human cytomegalovirus retinitis of the left eye involving the superior temporal arcade and parts of the posterior pole and the macula. *C*, Detailed image of retinitis of the right eye. *D*, Tomography of the macular region of the right eye demonstrating its preserved thickness. *E*, Detailed image of retinitis of the left eye. *F*, Thinning and disorganization of the macular zone layers of the left eye, tomography.

**Figure 2:**
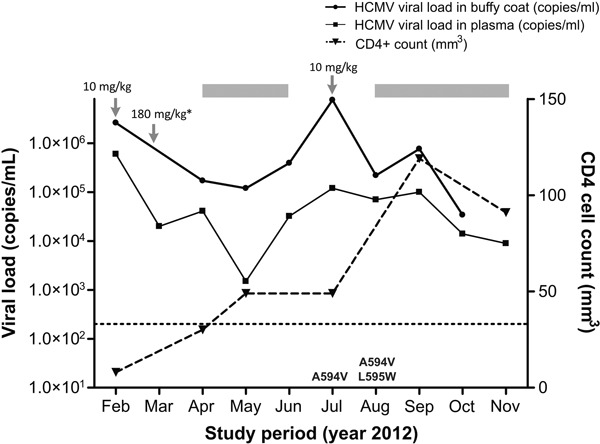
Changes in the human cytomegalovirus load (HCMV) (plasma and buffy coat) and the CD4^+^ T cell count over the study period. Gray bars: continuous treatment with 5 mg/kg daily ganciclovir *iv*. Changes in the applied dose of ganciclovir are indicated at February and July. The ganciclovir resistance mutations are indicated at the bottom of the graph. *iv foscarnet.

In February 2012, the patient complained of decreased visual acuity and blurred vision. Eye examination using tracking laser tomography (Spectralis, Heidelberg Engineering Inc., Germany) revealed a typical presentation of HCMV bilateral retinitis, characterized by focal hemorrhages, exudates in both eyes, and thinning, and disorganization of the retinal layers (Figure 1). At that time, the pp65 antigenemia test indicated 1 infected cell/2×10^5^ leukocytes, and the CD4^+^ cell count was 8 cells/mm^3^. Treatment with intravenous GCV (10 mg/kg daily) was initiated, and the oral ART regimen was changed (zidovudine, 600 mg/day; lamivudine, 300 mg/day; tenofovir, 300 mg/day; atazanavir, 300 mg/day; ritonavir, 100 mg/day). The HCMV treatment continued for 25 days but no clinical resolution of the ocular infection was observed (HCMV load, 1.2-3.9×10^5^ copies/mL); however, the HIV load was reduced to 292 copies/mL, and the CD4^+^ cell count increased to 30 cells/mm^3^. Because of the observed failure of the HCMV treatment, GCV was withdrawn and empirical treatment with foscarnet was started (180 mg/kg daily, *iv,* for 10 days). Interestingly, foscarnet treatment improved the patient's condition (cicatrization of the retinal lesions, Figure 1), but the HCMV load remained relatively stable both in the plasma and the buffy coat. After foscarnet treatment was suspended, GCV was continued until July 2012 at a dose of 5 mg/kg daily. In July, a new induction dose of 10 mg/kg daily was administered because of the consistently high viral load. In August 2012, HIV load became undetectable (<50 copies/mL) and CD4^+^ cell count increased to 119 cells/mm^3^. As a consequence of this, the dose of GCV was reduced to 5 mg/kg daily (Figure 2).

In February 2012, the patient complained of blurred vision, and both retinitis and GCV resistance were suspected. HCMV *UL97* was amplified and sequenced each month during the entire treatment period. Initially, no GCV resistance mutations were observed. However, after seven months of GCV treatment, the A594V mutation was detected. This mutation confers a GCV resistance ratio (effective concentration, EC_50_) ranging from 4.5 (12) to 10.4 mM (13). Despite the fact that the viral load had been reduced significantly by the eighth month (1.4×10^4^ copies/mL, plasma; 3.4×10^4^ copies/mL, buffy coat), another mutation related to GCV resistance (L595W) was detected, with a resistance ratio of EC_50_=5.1 mM (14).

## Methodology

Each month (from February to December 2012, 10 total samples), 6 mL of whole blood were collected in sterile EDTA tubes (Vacuette, Greiner Bio-One, Brazil). Viral (plasma) and cellular DNA (buffy coat) were extracted using a QIAamp Viral RNA mini kit (QIAGEN, Brazil), and a Gentra Puregene Purification Kit (QIAGEN), following the manufacturer's instructions. HCMV load was quantified in plasma and buffy coat using an in-house optimized TaqMan¯ real-time PCR assay, using the primers UL97F (5′-ACCGTCTGCGCGAATGTTA-3′) and UL97R (5′-TCGCAGATGAGCAGCTTCTC-3′), and the hydrolytic probe UL97P (5′-FAM-CACCCTGCTTTCCGAC-3′ MGB). Standard real-time amplification conditions were applied (initial activation step of 50°C/5 min, denaturation of 95°C/10 min and 40 cycles of denaturation at 95°C/30 s and a combined annealing and elongation step at 60°C for 1 min). The reaction was performed in a 25 µL final volume including 250 nM of each primer and 100 nM of the probe. The detection limit of the reaction was 6.9 copies/reaction (95% confidence interval). For *UL97* genotyping, a 1193-bp fragment was amplified and sequenced. The reaction was performed as a nested PCR and the initial larger fragment was amplified using the outer primer pair 803F (5′-ACGACGTGCATTGCACCTGTTC-3′)/1996R (5′-ACCATGCTGCACGAATACGTCA-3′). The nested PCR was performed using two internal primer pairs: UL97rF (5′-AGTGTCGTGTATGCCACTTTGA-3′)/UL97rR (5′-TGCGAGCATTCGTGGTAGAA-3′) and 833F (5′-AGATCATCACCACGTCCATCCGC-3′)/1457 R (5′-TCGCTGAGGCTGTAATCGCACA-3′), which amplified fragments of 624 and 627 bp, respectively. The amplification protocol for the first reaction included 10 initial cycles consisting of denaturation at 95°C/30s, annealing at 55°/30s and elongation at 72°/2 min. This stage was followed by 20 cycles consisting of denaturation at 95°C/30s, annealing at 55°C/30s, and elongation at 72°C/2 min. The elongation time was increased gradually by the addition of 20 s increment to each step. The second reaction consisted of initial denaturation at 95°C/5 min and 40 cycles of denaturation at 95°C/1 min, annealing at 58°C (primer pair 833F/1457R) or 60°C (primer pair UL97rF/UL97rR)/1 min and elongation at 72°/1 min 30 s. All the reactions were performed in 50 µL final volume.

DNA sequencing was performed using an ABI 3500XL genetic analyzer (Life Technologies, USA) and a Big Dye Terminator Sequencing kit (v3.1; Life Technologies, Brazil) following the manufacturer's instructions. The obtained *UL97* sequences were analyzed for the presence of GCV resistance mutations using software used for detection of drug resistance, available at <http://www.informatik.uni-ulm.de/ni/mitarbeiter/HKestler/hcmv/> (University of Ulm, Germany) (15).

## Discussion

Active HCMV infection in patients living with HIV shows generally stable initial viral loads, and its treatment with specific anti-HCMV drugs (mainly GCV), in association with strong adherence to ART, generally leads to a reduction in HCMV plasma levels (16). However, in the present case, despite continuous treatment with GCV and the inclusion of foscarnet in the therapeutic regimen, HCMV load remained relatively stable (Figure 2). During follow-up at the AIDS Clinic, the patient developed bilateral HCMV retinitis (Figure 1) that was non-responsive to GCV and was only influenced by foscarnet administration and an increase in the CD4^+^ cell count. It is possible that the increase in CD4^+^ cell numbers was largely responsible for healing the retinal lesions, which also explains the continuously high HCMV load (slow immunologic reconstitution). At the time of writing, the patient was receiving tenofovir disoproxil fumarate, lamivudine, raltegravir, maraviroc, darunavir, and ritonavir because of HIV resistance to nucleoside, non-nucleoside reverse transcriptase, and protease inhibitors.

Genotyping of all HCMV-positive samples obtained monthly during the GCV/foscarnet therapy period (10 months) revealed the emergence of two mutations related to GCV resistance: A594V and L595W in *UL97*. The emergence of A594V was observed after ∼240 days of treatment, while L595W appeared one month later. This is consistent with other studies examining HCMV resistance in patients with AIDS, which demonstrate an approximate period of 7 months for the appearance of resistance mutations (17). Interestingly, in this case the second mutation (L595W) appeared shortly after the first, and may be explained by a higher evolutionary pressure exerted on this gene by the GCV doses used and the continuous treatment period.

Surprisingly, the initial GCV treatment did not reduce the viral load, although resistance mutations were not detected. On the one hand, higher viral load at the time of GCV initiation could have negatively influenced the treatment outcome (18,19). At the time of the diagnosis of HCMV retinitis, the patient had a high viral load, which may have contributed to the observed negative response to treatment. On the other hand, other factors, including profound immunosuppression, previous exposure to antiviral drugs, and persistently low levels of HCMV DNA, are also thought to be responsible for the failure to treat HCMV (6). Of note, at the beginning of GCV treatment, the CD4^+^ cell count was 8 CD4^+^ cells/mm^3^. Therefore, we believe that the initial failure to treat the HCMV infection in this patient was caused by a combination of several factors, including a high HCMV load and profound immunosuppression. A low serum concentration of both drugs as a possible risk factor for the emergence of HCMV resistance or failure of HCMV treatment is unlikely in this case, as all of the antivirals (GCV and foscarnet) were administered in full treatment doses and not as a preemptive therapy.

The A594V mutation is one of the most common mutations related to GCV resistance, and, along with some other mutations (M460V, M460I, H520Q, C592G), is found in 90% of HCMV-resistance cases (20). In general, this mutation does not confer high levels of GCV resistance (three- to five-fold increase in 50% effective concentration value, EC_50_). It was described in 1997 by Smith et al. (2), with a reported resistance ratio of 14.0 µM. However, other studies demonstrate even lower rates of resistance, ranging from 3.9 (12) to 8 µM (20). The second observed mutation (L595W) in the current patient, which emerged one month later, is a rare mutation (14), and confers a resistance ratio of 15 µM. Because these two mutations appeared consecutively and quite late in the patient management process, we believe that their role in the initial treatment failure was minimal, but their combined effects on resistance may have induced a higher level of GCV resistance than observed individually.

Confirmation of the true GCV resistance ratio of any detected clinical isolate requires a phenotypic assay. However, this process is slow (can take months) and has specific laboratory requirements. The software used in the current study gives an approximate estimation of the GCV resistance ratio, and can distinguish between viral polymorphisms and resistance mutations. Therefore, a relative phenotypic image (virtual phenotype) could be deduced from the obtained sequence data in just a few hours, which can have important consequences for the management of an HIV-infected patient with opportunistic HCMV infection.

Although consecutive resistance mutations appeared after ∼240 days of GCV treatment, we believe that the success of the anti-HCMV treatment also depends on treatment of the HIV infection and tight monitoring of the reconstitution of the patient's cellular immune response. The mutations in this case had secondary role in treatment outcome and conferred only low-level GCV resistance. We believe that discussion of this clinical case will add to our understanding of the kinetics of the emergence of GCV resistance mutations in patients with AIDS and HCMV-related diseases.
